# Innovative temporary edge protection system in trench works for the construction industry and rescue operations

**DOI:** 10.1371/journal.pone.0283319

**Published:** 2023-03-23

**Authors:** Luis Miguel Ortiz García-Minguillán, José Tejero Manzanares, Rafael Enrique Hidalgo Fernández, Xiaoxin Zhang

**Affiliations:** 1 Departamento de Expresión Gráfica, Universidad de Córdoba, E.T.S.I.A.M. Córdoba, Córdoba, Spain; 2 Departamento de Mecánica Aplicada e Ingeniería de Proyectos, Universidad de Castilla-La Mancha, E.I.M.I. Almadén, Almadén, Ciudad Real, Spain; University of Vigo, SPAIN

## Abstract

This study assessed an innovative temporary edge protection system (TEPS) designed and developed to improve the ergonomics, health and safety at trench works in the construction industry, which ranks as one of the most hazardous worldwide. A wooden prototype of this innovative TEPS was built, and its mechanical resistance was compared to a conventional one. Thereafter, field tests were carried out to evaluate the efficacy of this pioneering TEPS. Both in analytical studies and field tests, the mechanical resistance to static loads of the TEPS complied with EU standards, and outperformed the conventional TEPS. The novel TEPS is effective as fall protection for different trench shoring systems and in buildings without edge protection systems. Moreover, this novel lightweight TEPS is easy to transport, with simple and safe on-site installation.

## Introduction

The construction industry is ranked as one of the most hazardous activities worldwide [1–6]. It accounted for the highest number of fatal accidents in the European Union in 2015, and was responsible for one out of five fatal accidents [7]. Falls from heights were the leading cause of death, and the third cause of non-fatal lesions in the construction industry [8, 9], with 6858 falls from height fatalities being recorded in the United States from 1992 to 2010.

In order to prevent accidents, existing or potential hazards must be identified and eliminated [10, 11], by implementing preventive measures designed at each construction stage under the coordinated planning and competent supervision of engineers and skilled workers [12], and installing protection systems to the structure of the building or installing collective safety protections.

In regulatory terms, there is an extensive range of international standards and regulations limiting falls from height [13–16]. Moreover, a number of studies have shown that a TEPS is an excellent collective protection system that prevents a substantial number of falls from height accidents [17–20]. However, several static load and impact studies evaluating the performance of several TEPS [21–26] have found certain conventional TEPS failed to meet the international standards and specifications on the geometric and dynamic requirements of TEPS [27–31]. It should be noted that these standards and specifications vary considerably worldwide [32].

In building and construction projects involving work inside trenches or around the perimeters of an excavation, besides the hazard of falls from heights, workers are exposed to other fatal risks such as cave-ins. In order to prevent cave-ins, when stabilizing the trench with slope measure is not feasible, trench shoring and edge protection must be in place in excavations 1.30 meters or more in depth [33, 34]. Due to such reasons, it is essential to carry out a geotechnical study of the terrain to determine its characteristics together with the depth of the trench and the loads to which the excavation is subjected, as well as to determine the ideal method of shoring to avoid the collapse of the trench before to start the work involved in an excavation.

**Fig 1:**
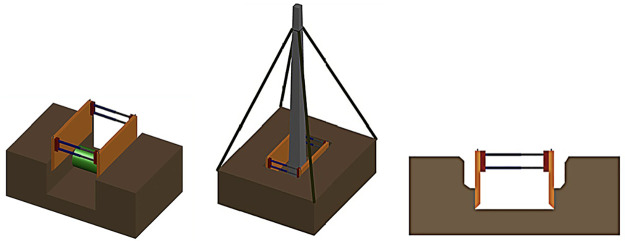
Examples of situations where prefabricated trench shoring systems are not feasible.

Currently, there is a wide array of metal trench shoring systems regulated by specific standards according to the methods employed [35–37]. These metal trench shoring systems have fixtures for facilitating transport, installation and removal. However, such systems entail certain drawbacks [38–40]:

- Most of these trench shoring systems are not feasible in trench sections crossed by underground utilities such as water, sewer, or gas pipes, and electric and telephone cables that obstruct the panels of the trench shoring system from resting on the excavation floor (see Fig 1a).
- These trench shoring systems cannot be used for installing utility poles such as power line posts as the erection and provisional support system uses a winch with four guide ropes, which obstruct the removal of the trench shoring system that would have to remain fixed in the concrete foundation (see Fig 1b).
- The geometry and assembly of prefabricated members of these systems limit their application to all situations.
- Generally, when the trench shoring system is placed inside the trench, there is a need to fill the gap between the forms and the excavation wall to ensure the adequate contact between both (see Fig 1c).

**Fig 2:**
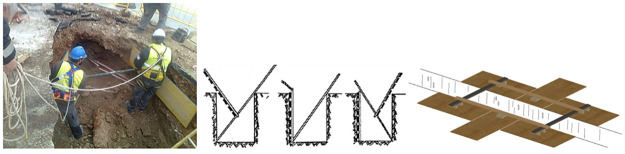
Traditional wooden trench shoring. (a) Traditional wooden trench shoring. (b) Quillery method. (c) Lip Bridges method.

In these cases, wooden trench shoring methods are used instead [6, 41], which are ineffective against water, costly in labour, and slow to install. Too often and incorrectly, workers enter the trench for installation of the shoring system which raises the risk of workers being buried by cave-ins during trench shoring works, as shown in Fig 2a. To avoid this danger, there are different methods of wooden shoring system such as Quillery or Lip Bridges allow the trench shoring work to be performed from outside but do not prevent the worker from falling into the trench, see Fig 2b and 2c.

**Fig 3:**
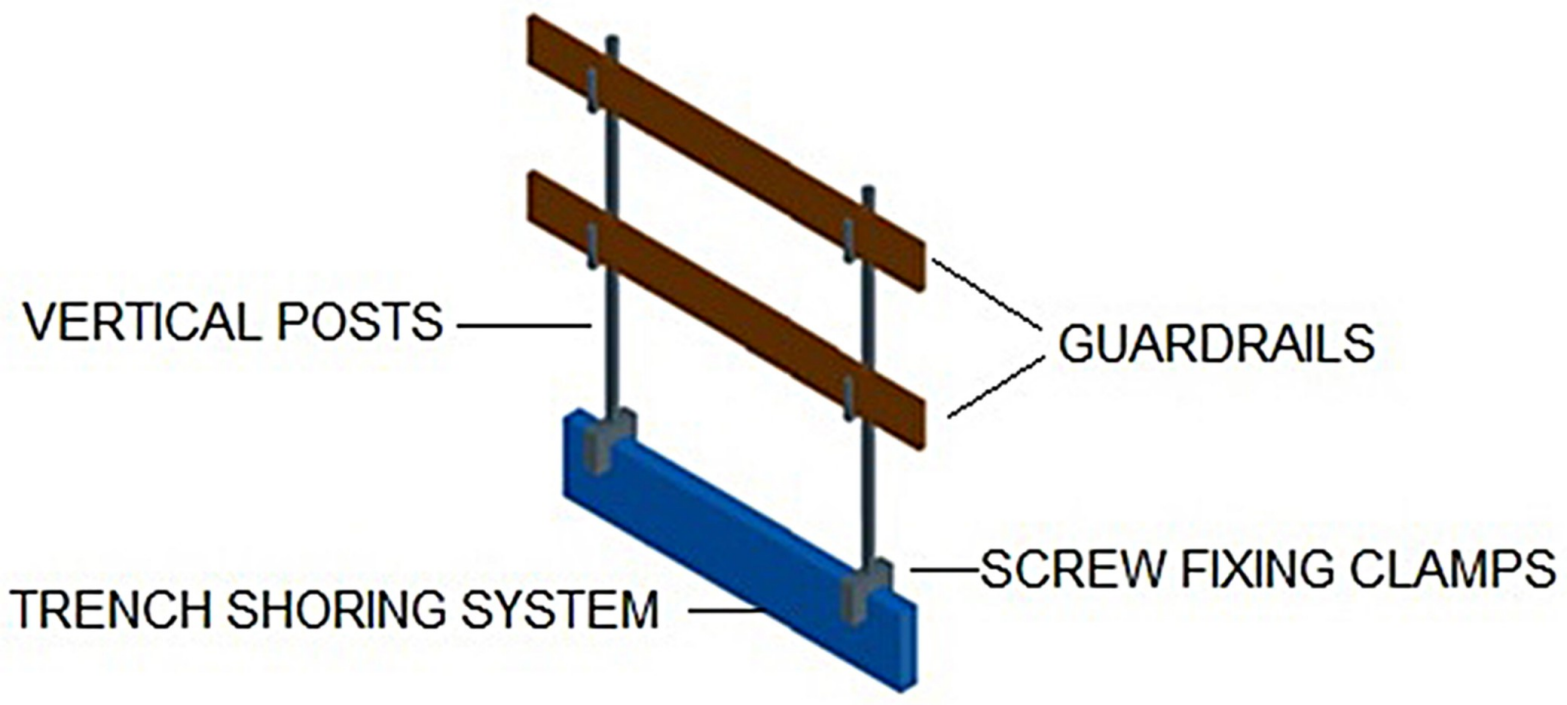
Conventional TEPS.

Moreover, metal trench shoring systems do not provide a protective barrier to help prevent falls from height into excavations. This underscores the need to supplement trench shoring systems with a TEPS. Conventional TEPS consist of vertical posts attached to the trench shoring systems using screw fixing clamps that hold the horizontal guardrails preventing workers from falling into trenches, as shown in Fig 3. For the correct installation of the TEPS, the trench shoring panel must protrude outwards at least 15 cm from the top edge of the trench wall.

Conventional TEPS have two shortcomings: first, as these TEPS are not fully integrated and all of the components have to be assembled at the edge of the trench, if the excavation is two meters or more in depth, the installation of the TEPS must be considered a work in height, and fall arrest systems must be in place; second, when force is applied to the top of the barrier posts, the maximum bending moment and shear force are generated at the base, which often fail to comply with the minimum bending, or mechanical strength requirements.

Bearing in mind these shortcomings, the aim of this study is to design and develop an innovative lightweight, temporary edge protection system for trench works, which is easy to transport, simple and safe to install, consisting of a fall protection barrier to prevent workers and objects from falling into trenches. Moreover, this novel edge protection system is valid to prevent people and objects from falling in buildings that do not have railings on their platforms. Furthermore, it is also valid for rescue missions, due to the fact that even rescuers are properly trained, they suffer falls during rescue operations sometimes. In addition to the structural requirements regulated by existing standards and specifications for the design of this novel TEPS, field studies with professionals, and interviews with experts were carried out in order to assess the systems efficacy, safety, and flexibility, as recommended by several studies [42, 43].

It is worth noting that small construction companies overlook health and safety standards, and are driven by competitive cost, completion time constraints and deadlines imposed by the construction industry, so the risk of falls from height is taken as “part of the job”, and the control of the potential danger of a fall from height is perceived as each worker’s own responsibility [44]. This underscores the need for heightening awareness and training for both construction companies and their workers to ensure the successful and efficacious implementation of prevention health and safety measures [45]. Such concerns can be solved by the innovative TEPS as well. Moreover, the innovative TEPS prototype was exhibited at a trade fair on health and safety at work held at the installations of the electricity company, Unión Fenosa Distribución, which is a part of the Naturgy group, and at a technical exhibition of the Mining and Industrial Engineering School of Almadén, Spain.

## Conceptual design

As stipulated in the standard EN-13374 [27], a TEPS is a set of components designed to protect people and objects from falling to a lower level. According to the height of the fall and the slope of the work surface, there are three different classes of TEPS (A, B, C). A Class A TEPS provides protection for flat and inclined surfaces less than 10 degrees with no limit in height of fall. A Class B TEPS provides protection for flat and inclined surfaces up to an angle of 30° with no limit in height of fall. For surfaces with a 30º to 60º inclination there is a 2 m limit in the fall of height. A Class C TEPS provides protection for a work surface inclination of 30º to 45º without limitation of the falling height. For surfaces with a 45º to 60º inclination there is a 5 m limit in the fall of height.

The TEPS assessed in this study is Class A that is designed to withstand only static loads, based on a requirement to: Support a person leaning on the protection or provide a handhold when walking beside it, and stop a person who is walking or falling towards the protection.

Moreover, Class A TEPS must comply with other basic requirements specifying the characteristics of the components that compose the system and its design.

- A TEPS consists of a top guardrail and an intermediate guardrail or an intermediate protection, to which a plinth can be attached, and all components in the system shall be designed to avoid accidental removal or displacement of any component in any direction during use.
- The distance between the top guardrail and the work surface is at least 1000 mm, as measured at any point perpendicular to the work surface. The top guardrail shall be continuous, and no horizontal space shall be wider than 120 mm.
- The distance between the highest part of the plinth and the work surface shall be at least 150 mm, as measured at any point perpendicular to the work surface.
- The plinth shall be designed to prevent gaps between the plinth and the work surface. A sphere with a diameter of 20 mm should not pass through them.
- The maximum vertical is up to 15º either inwards or outwards.

### Innovative (New) TEPS design

**Fig 4:**
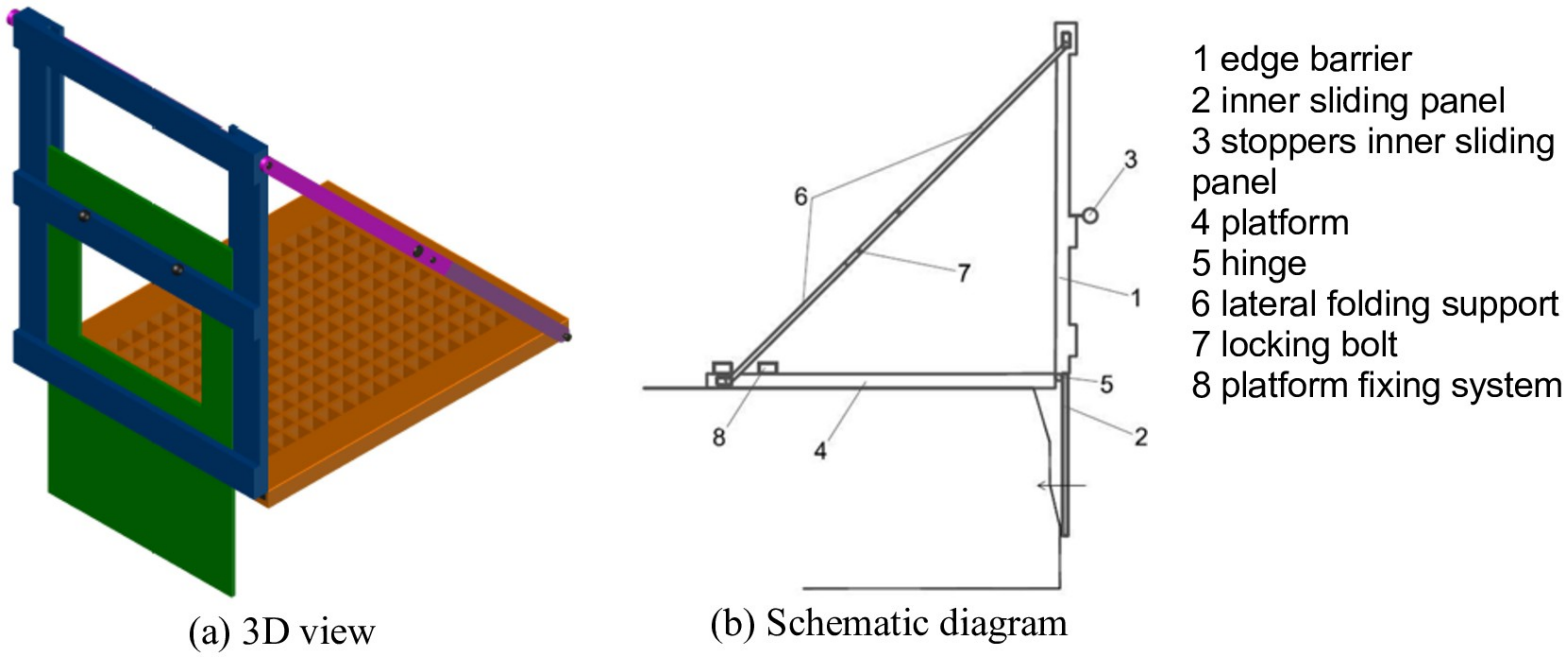
TEPS prototype. (a) 3D view. (b) Schematic diagram.

To prevent workers and objects from falling into a trench with shoring system and without shoring system but with a depth of less than 1.30 meters and cohesive soil, an innovative TEPS has been designed, see Fig 4, which can be used in cohesive soils. In the reference [46], defines the cohesive soil as the type of soil having plasticity whose proportion by weight of the content of clay (fine grains) is equal to or greater than 35%. Therefore, the cohesive soil contains small particles and enough clay for the soil to stick to itself. Moreover, it is necessary to carry out a geotechnical study of the soil to find out its characteristics and choose the ideal shoring system before starting excavation work. The TEPS consists of several components, the main body is an edge barrier that functions as a conventional protective barrier consisting three transversal guardrails attached to two vertical posts and that together compose an integrated TEPS. The edge barrier is attached to the platform by two hinges and two lateral folding supports to lift and maintain the edge barrier upright. Each lateral support is secured using a locking bolt when the edge barrier is at a 90º angle to the platform, this form reinforces the structural stability and rigidity.

The system has a sliding inner panel that runs on the guiderails along inner the main body, which enables trench shoring in the upper part of the trench wall and strengthens the resistance of the plinth of the TEPS. The platform provides the worker a stable working surface, and spreads the worker’s body weight throughout the entire platform, which considerably reduces the risk of trench collapse.

### Installation of the new TEPS

**Fig 5:**
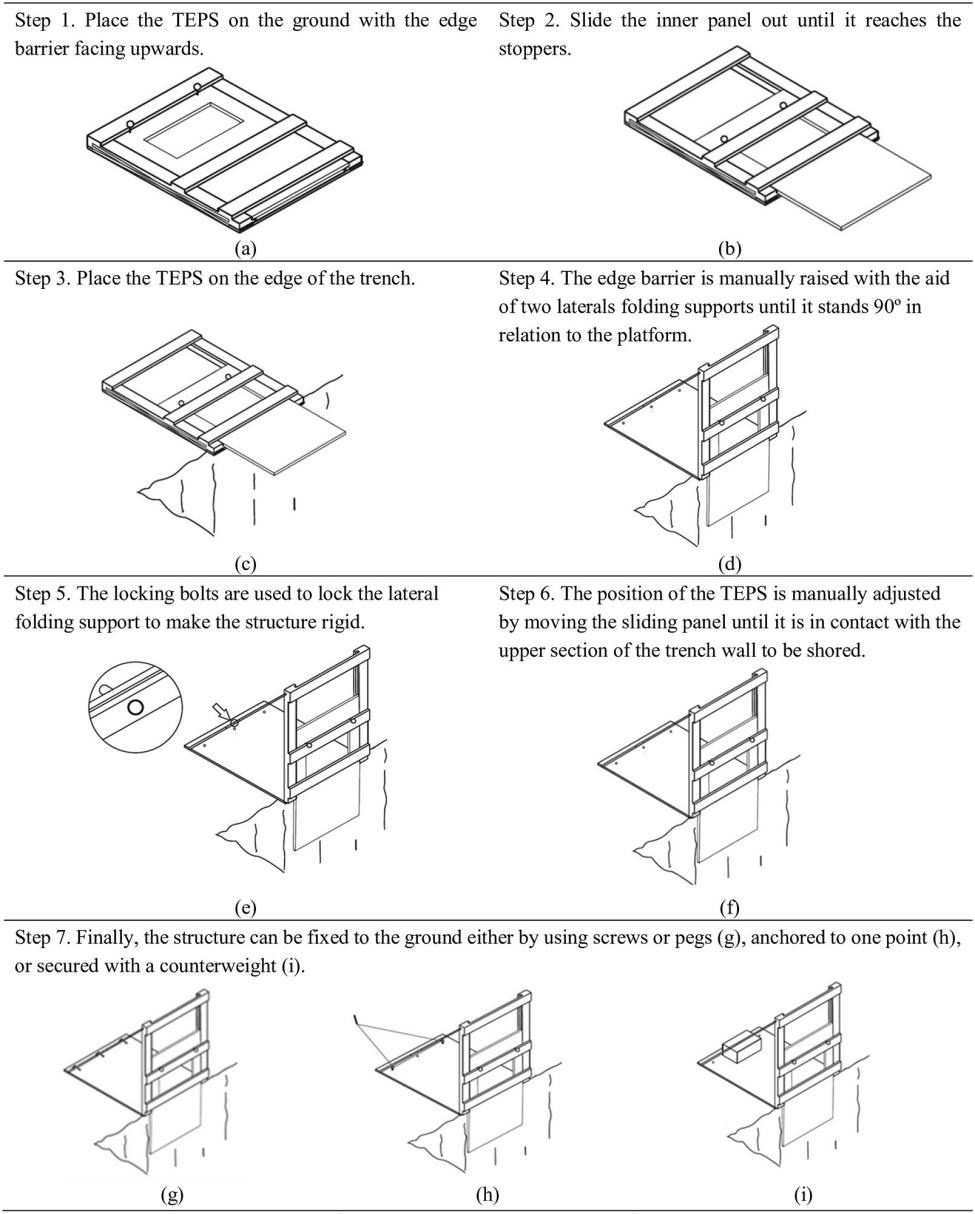
Typical TEPS assembly process.

This new TEPS is easy to transport, simple and safe to install with no need for workers to be in the trench. The process is described in Fig 5.

## Evaluation techniques

For assessing the efficacy of the system, a wooden prototype of the new TEPS was built. The values of the wooden members are listed in Table 1. The dimensions of both the guardrails are 800 mm x 120 mm x 22 mm. The dimensions of the posts of the protection barrier are 1000 mm x 95 mm x 22 mm, and 1000 mm x 80 mm x 18 mm, respectively. In accordance with the standards EN-338 [47] and EN-56544 [48], the wood used has the visual classification ME1 with a resistant class of C27, and the dimensions of the lateral supports are 850 mm x 30 mm x 15 mm, with a resistant class of T8.

**Table 1:** Characteristics of the wooden members of the TEPS prototype.

Elements	Guardrails	Posts		Lateral supports
Dimensions (*mm*)	800 x 120	1000 x 95	1000 x 80	850 x 30
Thickness (*mm*)	22	22	18	15
Visual class	ME1	ME1	ME1	ME1
Resistant class	C27	C27	C27	T8

**Fig 6:**
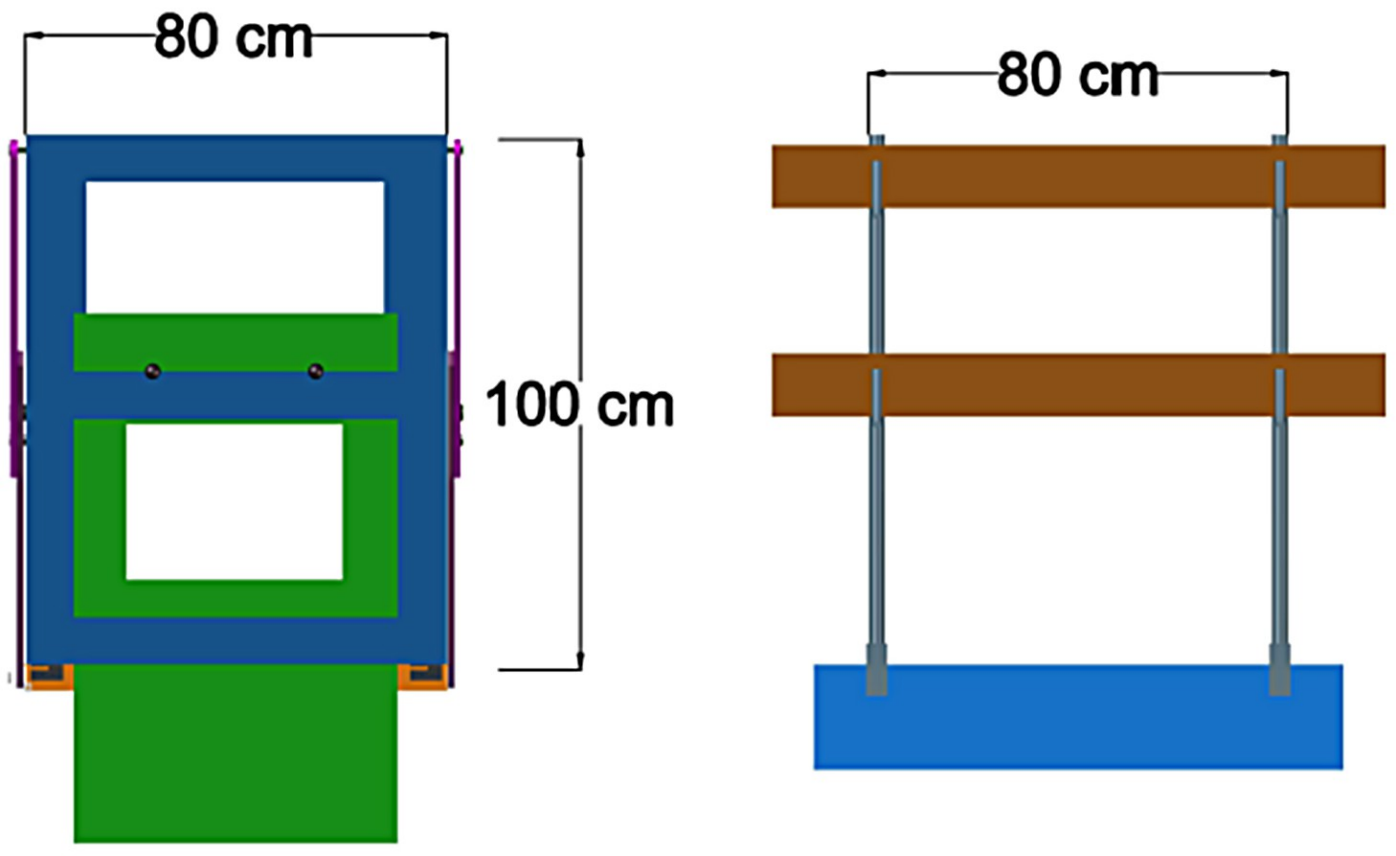
Schematic diagrams of the TEPS. (a) New TEPS prototype. (b) Conventional TEPS.

The geometric dimensions of the edge barrier are 100 cm high and 80 cm wide, with a top guardrail, intermediate one and bottom rail, as shown in Fig 6a.

In the following section, the calculations of the mechanical resistance of the new TEPS were compared with a conventional one (See Fig 6b) consisting of two vertical posts crossed by two horizontal wooden walers, one at the top and the other at the centre of the post, the posts are 100 cm high and 80 cm apart.

### Types of analysis

As for the calculations performed on this Class A TEPS system used on flat surfaces and slopes up to 15º in compliance with the standard EN-13374 [27], the assessment of the mechanical properties of the materials used in the TEPS prototype under static loads analysed three critical situations: a) Ultimate Limit State (ULS) with fundamental load. b) Ultimate Limit State with Accidental Load (AL), and c) Serviceability Limit State (SLS). All three situations imply

$$E_{d}\leq R_{d}$$ (1)

where,

*E*_*d*_: effect of the actions

*R*_*d*_: structural mechanical resistance

For the assessment of the new TEPS prototype, the guardrails and posts forming the structure of the edge barrier were analysed separately. The same procedure was applied for the assessment of the conventional TEPS.

The EN-13374 standard [27] for ULS specifies that guardrails or vertical barrier posts shall be designed to withstand a load of 0.30 *kN* applied perpendicularly to the plane of system at the weakest point. For the evaluation of this limit state, a coefficient of increasing resistance (γ_F_) with a value of 1.5 for all of the loads, and a coefficient of decreasing resistance (γ_M_) of the material was applied, which had a value of 1.3 for wood. With these figures, the load applied to each wooden structural component was obtained in Eq (2).


$$F_{H1}=0.30•\gamma_{F}•\gamma_{M}=0.30•1.5•1.3=0.6\;kN$$ (2)

In order to satisfy the SLS, the deflection (δ) should be no greater than 55 mm when a single horizontal load of *F*_*H*_ = 0.30 *kN* was applied to at the weakest point of the system.

In the case of AL, guardrails should withstand a single gravitational load of 1.25 *kN* applied to the weakest point of the TEPS, on an inclined slope ± 10° in relation to the vertical plane. In order to evaluate this limit state, a coefficient of increasing resistance (*γ*_*F*_) with a value of 1 for all of the loads, and a coefficient of decreasing resistance (*γ*_*M*_) of the material was applied, which has a value of 1.3 for wood. Bearing these figures, the load applied to each wooden structural component was determined in Eq (3).


$$F_{D}=1.25•\gamma_{F}•\gamma_{M}=1.25•1•1.3=1.62\;kN$$ (3)

### Comparison between the conventional TEPS and the innovative one

The calculus model adopted for the analytical evaluation of the guardrails consisted in them being considered as beams, the ends of which rest on two support points.

In the case of the barrier posts, the calculation model for the analytical evaluation of the conventional TEPS bore in mind the bottom end of the post was imbedded in the soil. For the analytical evaluation of the novel TEPS, it was considered to be anchored to a bi-articulated support at the bottom and fixed to at the top to the lateral post.

#### ULS analysis

**Fig 7:**
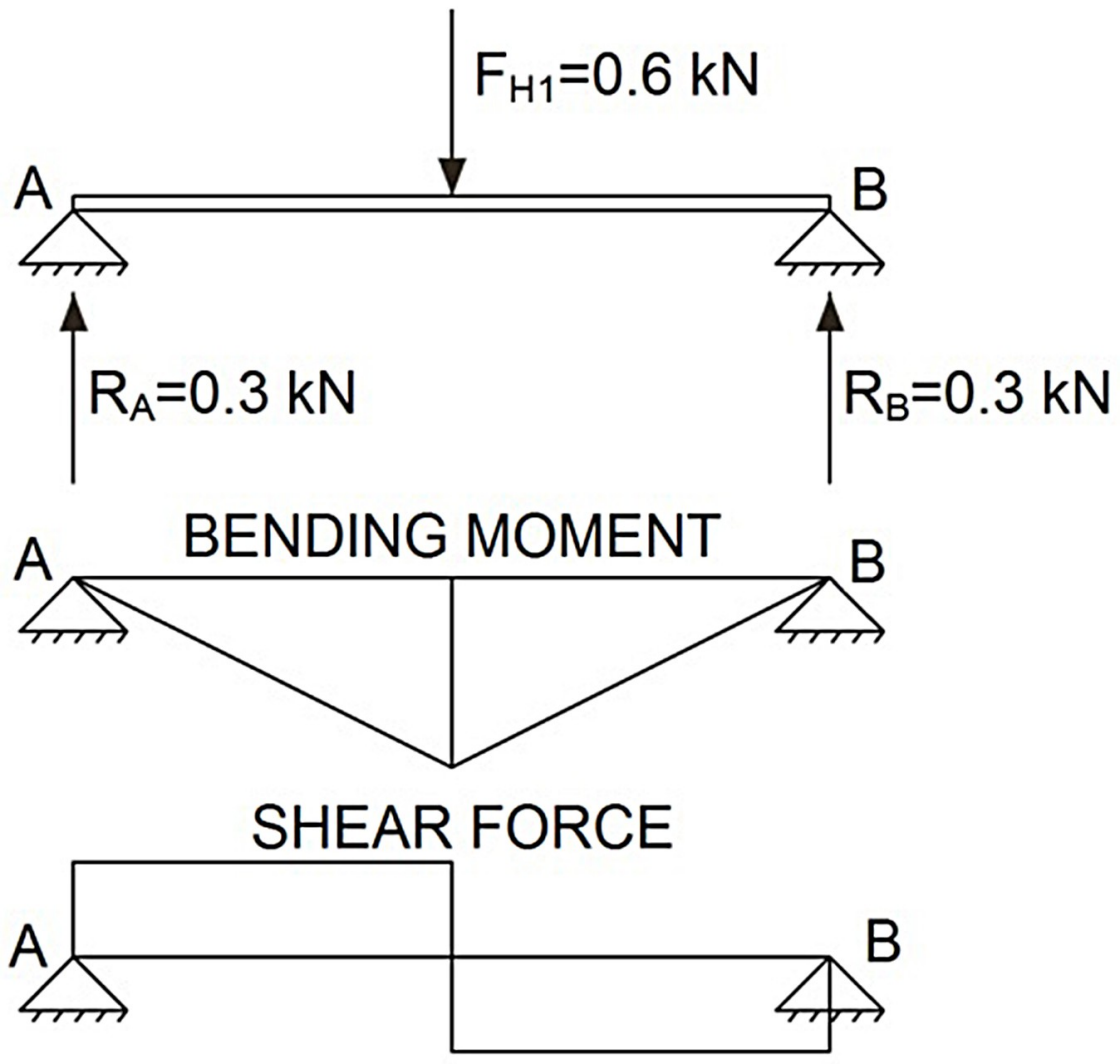
ULS on guardrails.

The same ULS analysis was used for both the top guardrail and the intermediate guardrail. The most unfavourable situation for the members of both TEPS was when the load was applied to the centre of the guardrail (maximum moment point), see Fig 7.

**Fig 8:**
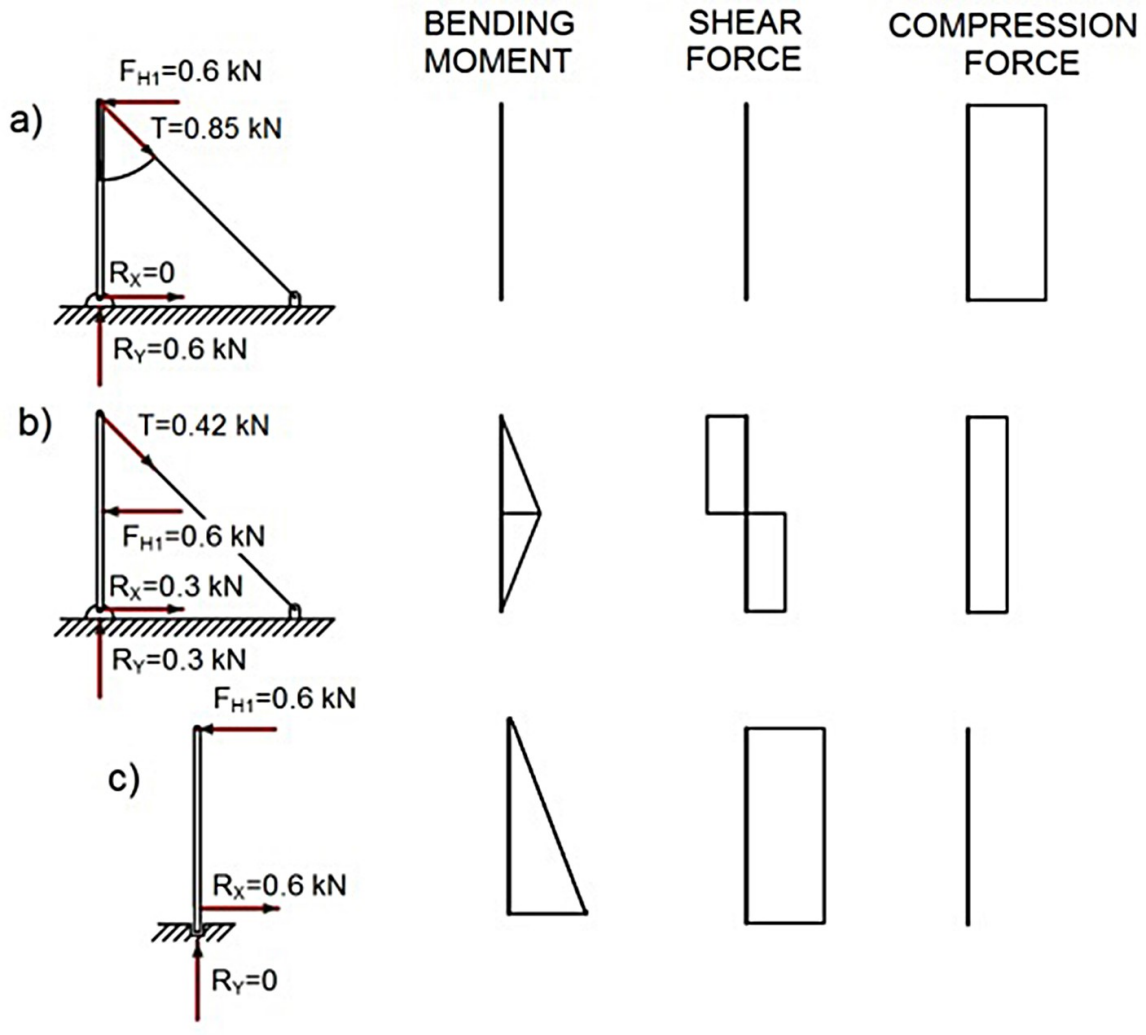
ULS on posts.

In the case of the posts, the most unfavourable situation for the conventional TEPS was when the load was applied to the edge of the cantilever, the bottom of post being the most unfavourable section, where the bending moment and the maximum shear force were produced, as shown in Fig 8c.

However, when a transversal force was applied to the top of the post of the TEPS prototype, the lateral posts counteracted the transversal force and the moment generated by it, producing in the lateral posts a longitudinal traction force of 0.85 *kN* (*F*_*H1*_/sin 45º = 0.85 *kN*), and a compression force on the post with a value similar to that applied to the top of the post, see Fig 8a.

When this transversal force was applied to the centre of the post of the TEPS prototype, a bending moment and a longitudinal compression force of 0.3 *kN* was generated on the post, as shown in Fig 8b. In the lateral posts, a traction force slightly above 0.4 *kN* was obtained:

#### AL analysis

For the calculation of the accidental actions on the guardrail, the same methodology as in the ULS calculation process was applied, but a vertical load *F*_*D*_ = 1.62 *kN* was applied at the most unfavourable point.

**Fig 9:**
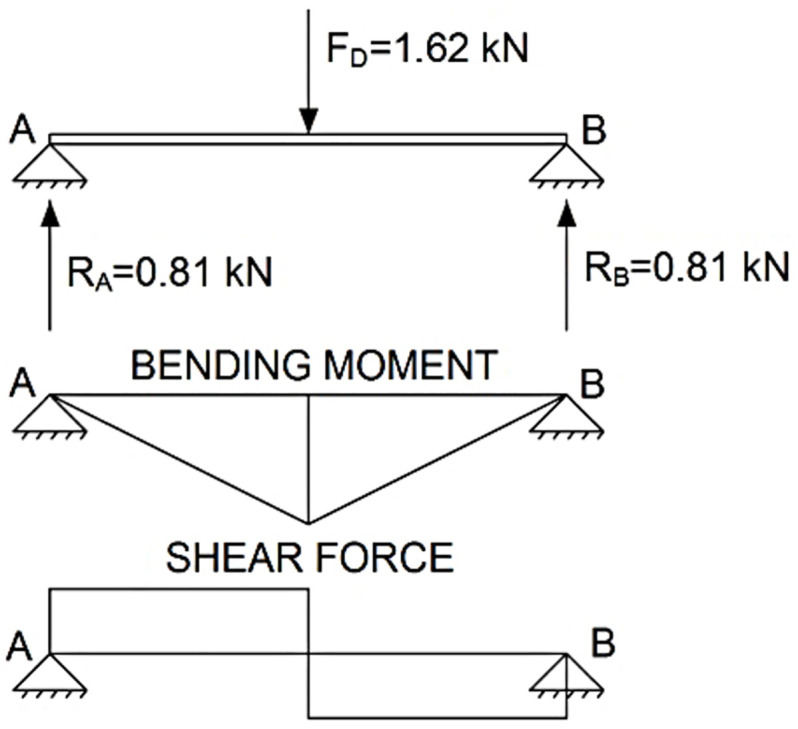
AL analysis on guardrails.


The analysis was identical for both the top and intermediate guardrails. The most unfavourable point of these elements was when the load was applied to the central point of the rail, and the maximum bending moment was obtained in this section of the rail, see Fig 9.

$$\text{Maximum}\;\text{bending}\;\text{moment}=\left(F_{D}•d\right)/4=324\;kN•mm,\;d\;\text{is}\;\text{the}\;\text{span}\;800\;\text{mm}$$ (4)

$$\text{Maximum}\;\text{shear}\;\text{force}=F_{D}/2=0.81\;kN$$ (5)

**Fig 10:**
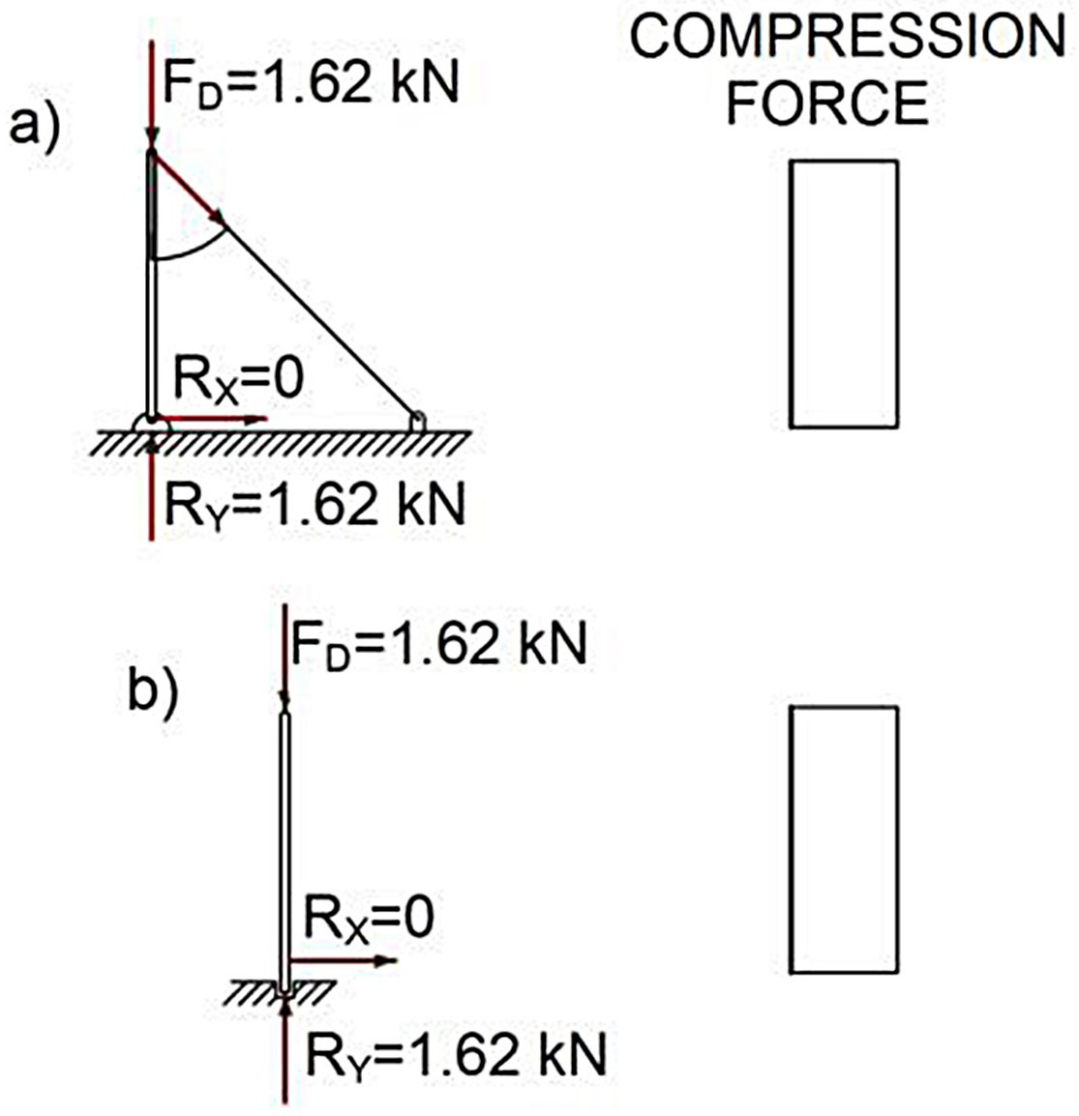
AL analysis on posts.

As for the posts, when gravitational force was applied to the top of the posts with an inclination of 0º, a similar longitudinal compression force was produced in both TEPS, given that in the TEPS prototype the tension produced in the lateral supports was 0, see Fig 10.

For a better comparison, the results of the ULS and AL analyses for the conventional TEPS and new TEPS (prototype) were presented in Table 2. It was observed that the analysis of both TEPS showed same results when the load applied on the top and intermediate guardrails. In contrast, the results for the posts showed the TEPS prototype outperformed the conventional one when the transversal force applied on the top and center of the post. For example, when transversal force was applied on the top of the post of the conventional TEPS, a bending moment of 600 *kN∙mm* was generated. In comparison, when the same transversal force was applied to the top of the posts of the TEPS prototype, the lateral posts neutralized this transversal force, and the moment generated by it. Moreover, when the transversal force was applied to the centre of the posts of the conventional TEPS, the result of the bending moment generated by the said force was twice as high as that generated in the TEPS prototype.

**Table 2:** Comparison between the results of the ULS and AL analysis for both TEPS.

Element and point where load was applied	Type of TEPS	Type of analysis			
		ULS		AL	
		Maximum bending moment (*kN•mm*)	Maximum shear force (*kN*)	Maximum bending moment (*kN•mm*)	Maximum shear force (*kN*)
Middle of top and intermediate guardrails	Conventional	(*F*_*H1*_*•d*_*1*_)/4 = 120	*F*_*H1*_/2 = 0.3	(*F*_*D*_*•d*_*1*_)/4 = 324	*F*_*D*_/2 = 0.81
	Prototype	(*F*_*H1*_*•d*_*1*_)/4 = 120	*F*_*H1*_/2 = 0.3	(*F*_*D*_*•d*_*1*_)/4 = 324	*F*_*D*_/2 = 0.81
Top of post	Conventional	*F*_*H1*_*•d*_*2*_ = 600	*F*_*H1*_ = 0.6	0	0
	Prototype	0	0	0	0
Central part of post	Conventional	*F*_*H1*_*•d*_*2*_/2 = 300	*F*_*H1*_ = 0.6	0	-
	Prototype	(*F*_*H1*_*•d*_*2*_)/4 = 150	*F*_*H1*_/2 = 0.3	0	-

Note: d_1_ = 800 *mm*, d_2_ = 1000 *mm*, *F*_*H1*_ = 0.6 *kN*, *F*_*D1*_ = 1.62 *kN*.

#### SLS analysis

**Fig 11:**
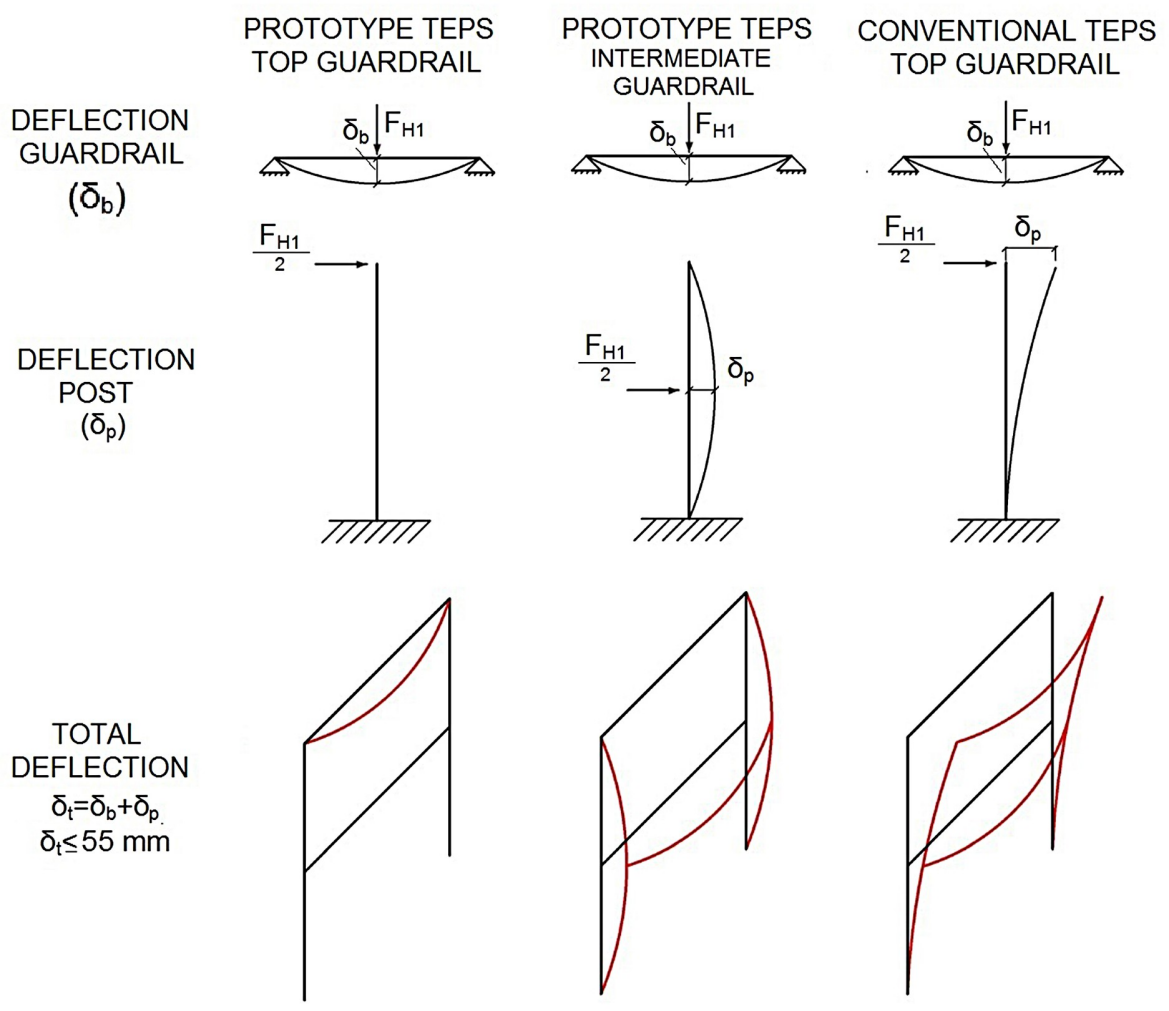
Analysis in SLS.

For the SLS calculations, the horizontal movement of the system, the deflection of the guardrail loaded in the central section, and the backward deflection of the post were evaluated. The deflection of the post was calculated with an action using half of the guardrail load applied at the edge, see Fig 11.

From Fig 10, it is clear, after applying the same load to the center of the top guardrail of both TEPS, deflection was observed to be greater in the conventional TEPS as compared to the TEPS prototype, given that deflection of the TEPS prototype post was almost zero. The standard indicates the sum of the deflection of both posts should be less than 55 mm.

## Validity and field tests of the TEPS prototype

On the basis of the calculations obtained, a wooden TEPS prototype was designed and fabricated. The guardrail dimensions are 800 mm x 120 mm x 22 mm. The guardrail and post dimensions are 1000 mm x 95 mm x 22 mm, and 1000 mm x 80 mm x 18 mm, respectively. All of the timbers used had the visual classification ME1 with a resistant class of C27. The dimensions of the wooden lateral supports are 850 mm x 30 mm x 15 mm, with a resistant Class of T8.

In order to determine the validity of both the Class resistance and the requirements of the wooden sections of the TEPS, several calculations were performed in line with the specifications detailed in the SE-M [49], considering the atmospheric conditions of serviceability, Class 3 (climatic conditions of higher atmospheric humidity than in Class 2, corresponding to a temperature of 20ºC and an relative atmospheric humidity that seldom exceeds 85% in the year), and a Class use of timber beams which are used for scaffolding.

### Limit states of the TEPS prototype

In order to ensure the specifications of the wooden members were right, according the specification in point 6 of the SE-M [49], which relating the stress and material strength for each load combination. For a section validated, the ratio between the stress and material strength should be less than one.

### Stress analysis in ULS

When the load was applied on the middle of the top guardrail, intermediate one and the top of post, the ratios for simple bending, and shear force of the elements were calculated and presented in Table 3. Moreover, for lateral supports, the ratio for traction force was also obtained. It is obvious that all the values are less than 1, meeting the requirement of the standard. In these cases, according the specification in point 6.1 of the SE-M [49], conditions must be fulfilled presented in Table 3.

**Table 3:** Values of the ULS for the TEPS prototype.

Type of force	Requirement	Element	Result
simple bending	*σ*_*m*,*d*_ *≤ f*_*m*,*d*_, *M*_*d*_*/(W•f*_*m*,*d*_*)* < 1	Guardrail	0.83
		Post	0.54
shear force	*τ*_*d*_ *≤ f*_*v*,*d*_, *(1*.*5•Q*_*d*_*)/(b•h•f*_*v*,*d*_*)* < 1	Guardrail	0.14
		Post	0.26
traction force	*σ*_*t*,*k*_ *≤ f*_*t*,,*k*_, *F*_*k*_*/(W•f*_*t*,*k*_*)* < 1	Lateral support	1•10^−4^

Note:

σ_m,d:_ designed bending stress. The maximum force in the section is *σ*_*max*_ *= M*_*d*_*/W*.

τ_d_: designed shear stress. The maximum tangential force occurs in the central fiber and its value is *τ*_*max*_ *= 1*.*5•(Qd/(b•h))*.

σ_t,k_: designed traction parallel to the fiber. Assuming that the force is distributed over the entire net surface *σ*_*t*,*k*_ *= F*_*k*_*/A*_*n*_.

M_d_: the maximum bending moment on the guardrails in ULS analysis as presented in Table 2.

W: the section modulus, *W = (b•h*^*2*^*)/6*, where b and h were the width and thickness of the section, respectively.

Q_d_: the maximum shear force on the guardrails as shown in Table 2.

F_k_: the traction force on the lateral support posts described in Section 3.2.1.

f_m,d_: the flexural strength of the material. 14.85 N/mm^2^ for guardrail, 13.068 N/mm^2^ for post.

f_v,d_: the shear strength of the material, 2.8 N/mm^2^ for both guardrail and post.

f_t,k_: the tensile strength of the material, 3.38 N/mm^2^.

When transversal force was applied to the centre of the post, combined axial bending and compression was generated at the centre. In these cases, according the specification in point 6.2.3 of the SE-M [49], the following conditions must be fulfilled:

$${\left(\sigma_{\text{c},0,\text{d}}/{\text{f}}_{\text{c},\text{o},\text{d}}\right)}^{2}+(\sigma_{\text{m},\text{y},\text{d}}/{\text{f}}_{\text{m},\text{y},\text{d}})+({\text{k}}_{\text{m}}•(\sigma_{\text{m},\text{z},\text{d}}/{\text{f}}_{\text{m}.\text{z}.\text{d}}))<1$$ (6)

$${\left(\sigma_{\text{c},0,\text{d}}/{\text{f}}_{\text{c},\text{o},\text{d}}\right)}^{2}+({\text{k}}_{\text{m}}•(\sigma_{\text{m},\text{y},\text{d}}/{\text{f}}_{\text{m}.\text{y}.\text{d}}))+(\sigma_{\text{m},\text{z},\text{d}}/{\text{f}}_{\text{m},\text{z},\text{d}})<1$$ (7)

where

σ_c,0,d_: strength calculation of parallel compression, *σ*_*c*,*0*,*d*_ *= Q*_*d*_*/A*_*n*,_ Q_d_ was the parallel compression force presented in Table 2, and A_n_ was the section area. In our case, the value is 0.25 *N/mm*^*2*^.

f_c,o,d_: calculation value of compression parallel strength of the material, 9.68 *N/mm*^*2*^.

σ_m,y,d_: flexural stress on the y axis, *σ*_*m*,*y*,*d*_ *= M*_*d*_*/W*, with M_d_ being the bending moment shown in Table 2, and W was the section

modulus. In our case, the value of the flexural stress is 7.03 *N/mm*^*2*^.

f_m,y,d_: calculation of flexural strength on the y axis, 11.88 *N/mm*^*2*^.

σ_m,z,d_: flexural stress on the z axis. In our case, the value is 0.

f_m.z.d_: calculation of flexural strength on the z axis.

k_m_: factor takes into account the effect of tension redistribution and the lack of homogeneity of the material of the transversal section, which in the rectangular solid wood sections, plywood, laminated wood and micro-laminated wood, the value was 0.7.

The posts were made of two planks: 1000 mm x 95 mm x 22 mm, and 1000 mm x 80 mm x 18 mm in dimensions, fixed together on the parallel fibre sides; thus, for the calculations they were considered laminated wood, Class resistant C27. According to the Eqs (8) and (9), it is obvious that the TEPS prototype meets the criterion of the standard, when transversal force was applied to the central point of the post.


$${\left(\sigma_{\text{c},0,\text{d}}/{\text{f}}_{\text{c},\text{o},\text{d}}\right)}^{2}+(\sigma_{\text{m},\text{y},\text{d}}/{\text{f}}_{\text{m},\text{y},\text{d}})+({\text{k}}_{\text{m}}•(\sigma_{\text{m},\text{z},\text{d}}/{\text{f}}_{\text{m}.\text{z}.\text{d}}))=0.59<1$$ (8)

$${\left(\sigma_{\text{c},0,\text{d}}/{\text{f}}_{\text{c},\text{o},\text{d}}\right)}^{2}+({\text{k}}_{\text{m}}•(\sigma_{\text{m},\text{y},\text{d}}/{\text{f}}_{\text{m}.\text{y}.\text{d}}))+(\sigma_{\text{m},\text{z},\text{d}}/{\text{f}}_{\text{m},\text{z},\text{d}})=0.41<1$$ (9)

### Stress analysis in AL

For the calculation of accidental actions on the guardrail, the same methodology was applied as in the ULS calculation process. Thus, ratios between the stress and material strength for simple bending and shear force were calculated on the guardrails. The ratio for compression force of the posts was obtained as well. The results of the stress analysis in AL are shown in Table 4. It is clear that all the values are less than 1, fulfilling the specification in point 6.1 of the SE-M [49], conditions must be fulfilled presented in Table 4.

**Table 4:** Values of the AL.

Type of force	Requirement	Element	Result
*simple bending*	*σ*_*m*,*d*_ *≤ f*_*m*,*d*_, *M*_*d*_*/(W•f*_*m*,*d*_*)* < 1	Guardrail	0.51
*shear force*	*τ*_*d*_ *≤ f*_*v*,*d*_, *(1*.*5•Q*_*d*_*)/(b•h•f*_*v*,*d*_*)* < 1	Guardrail	0.39
*compression force*	*σ*_*c*,*0*,*d*_ *≤ f*_*c*,*0*,*d*_, *N*_*d*_*/(A*_*n*_*• f*_*c*,*0*,*d*_*)* < 1	Post	0.05

Note:

σ_c,0,d_: designed compression stress parallel to the fiber. Assuming that the force is distributed over the entire net surface

*σ*_*c*,*0*,*d*_ *= N*_*d*_*/An*.

N_d_: the value of the load applied, 1.62 *kN*.

A_n_: the section area.

f_c,0,d_: the value of the calculated compression strength of the material.

### Verification of SLS deformation

The deformation of a beam with the load applied to the centre is determined by the Eq (10):

$$\delta =\left(P•L^{3}\right)/\left(48•E•I\right)$$ (10)

where *P* is the force applied to the beam, *L* stands for the beam span, *E* is the elastic modulus of the material and *I* is the moment of inertia of the section.

Accordingly, in the case of the guardrails, the deformation *δ*_*b*_ is 5.22 mm. For the two planks of the posts, the deformation *δ*_*p*_ is 2.54 mm. The total *δ*_*t*_ is 7.76 mm, which is less than 55 mm, the TEPS complies with the SLS requirements.

### Evaluation of the anchor system

Based on the forces which the system is going to stand, the depth of penetration of the anchors and their diameter should be checked.

**Fig 12:**
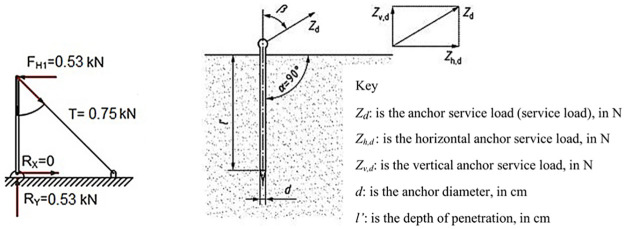
Check the capacity of the anchor. (a). (b) After EN 13782 [50].

As mentioned previously, for ULS analysis, the standard EN 13374 [27] specifies that guardrails or vertical barrier posts shall be designed to withstand a load of 0.30 *kN* applied perpendicularly to the plane of system at the weakest point. Moreover, following the standard EN 13782 [50], for the evaluation of anchor service load, two factors, 1.6 (coefficient of increasing resistance, γ_F_) and 1.1 (safety factor against overturning, sliding and lifting, γ) were used. Thus, the load applied to each structural component was obtained in Eq (11), see Fig 12a.


$$F_{H1}=0.30\;•\gamma_{F}•\gamma =0.30•1.6•1.1=0.53\;kN$$ (11)

When a transversal force was applied to the top of the TEPS prototype, the lateral posts counteracted the transversal force and the moment generated by it, producing in the lateral posts a longitudinal traction force of 0.75 kN ((F_H1_/sin 45º = 0.75 *kN*)), this force was also the anchor service load *Z*_*d*_, as shown in Fig 12b.

When the angle *β* equals 45ᵒ, as in our case, the load bearing capacity (*Z*_*d*_) of simple rod anchors with a circular cross section (diameter, *d*) and the depth of penetration (*l´*) can be determined by using the Eq (12) in accordance with the standard EN 13782 [50].

$$Z_{d}=f_{load}•d•l’=10•d•l’$$ (12)

where *f*_*load*_ is the coefficient, it is 10 for the cohesive soil.

Moreover, in order to prevent any bending of anchors subjected to oblique traction, the following diameter (Eq (13)) shall be kept for simple round steel rod anchors:

dmin=0.025•l’+0.5 (13)

Thus, if the diameter and depth of penetration are 1.8 *cm* and 45 *cm*, respectively, the load bearing capacity of each anchor will be 810 *N* (0.81 *kN*), which is over the traction force 750 *N* (0.75 *kN*). It is obvious that the system is safety. However, such calculations were made for the static loads, as a safety measure to support a possible dynamic accidental load to the system, three anchors with 100 *mm* spacing were used. It could resist 2.43 *kN* in total, which is also greater than the accidental load 1.62 *kN* mentioned in the Section “AL analysis”.

### Field studies

#### Static load test

In collaboration with a crew of workers from the Spanish electricity company: Electricidad Jesús Bárcenas, S.L., four field tests were carried out to assess the efficacy of the novel TEPS in several areas of the province of Ciudad Real (Spain). The crew’s main job was the erection, maintenance and repair of electricity poles in trenches up to two meters deep in cohesive terrain.

**Fig 13:**
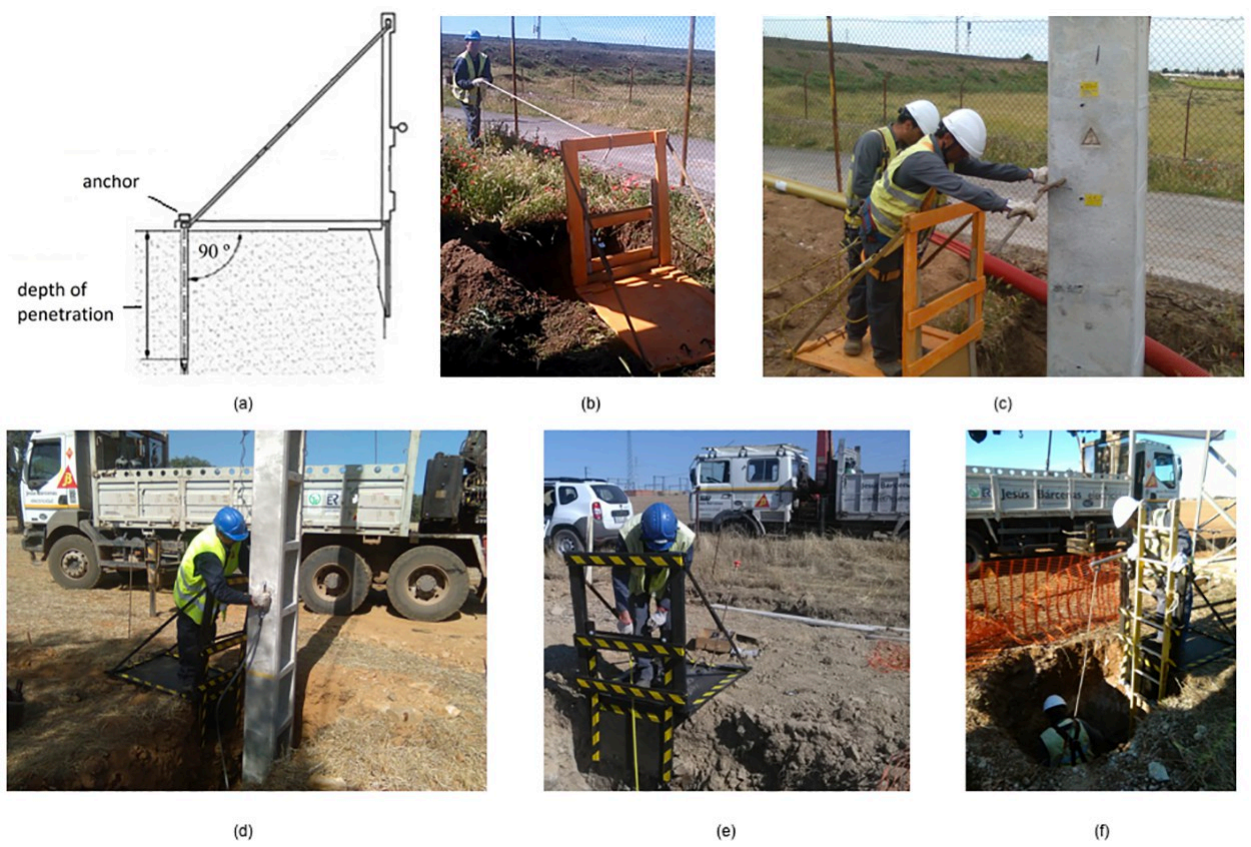
Static load tests.

According to the previous calculation, the TEPS was fixed by using three steel anchors, 450 mm in depth of penetration, and 18 mm in diameter, inserted with a 100 mm spacing in the rear end of the platform, the angle of penetration was 90º, as shown in Fig 13a.

After positioning and anchoring the TEPS, a resistance test was performed consisting of a worker pulling on a rope tied to the top guardrail for a short period, see Fig 13b. Based on kinesiology and biomechanical studies [51], the maximum stress exercised by a muscle depends on its transversal section, which in the case of men is from 0.3 to 0.4 N/mm^2^. Thus, a muscle of 1500 to 2000 mm^2^ is required to produce a muscle force of 600 N. As several muscles were involved simultaneously when the worker pulled on the rope, the force applied by the worker would be greater than 600 N.

Fig 13c-13f shows a crew of workers were positioning and erecting utility poles by using the TEPS prototype. All such field tests performed, no yielding, fracture, cracking, splintering or separation were found in the prototype. It shows that the TEPS prototype is fitful for the field operations.

#### Accidental load test

The protection system has been presented to both workers and experts from different construction sectors. In one of these evaluations, the suitability of the edge protection system for buildings that do not have railings on their platforms is suggested. Moreover, besides the TEPS field tests against static loads carried out, a TEPS test against dynamic loads was also performed in a rescue drill, due to the fact that even the rescuers are properly trained, they could suffer accidents on their rescue missions as well.

In collaboration with Mr. José Antonio Ruiz, Head Coordinator of Puertollano Civil Defence (Ciudad Real, Spain), a rescue operation was simulated on a rooftop using the TEPS prototype in order to assess its performance under an accidental load in height operations.

The tests were performed on the rooftop of the former Puertollano slaughterhouse. Among other elements, the material used in the simulated rescue operation consisted of a dummy, a spinal stretcher, with a total weight of 82 kg. In addition, a pulley was fixed to the top guardrail of TEPS aid the lifting and lowering of the stretcher in the operation.

Before carrying out the test, the impact force over the dummy itself in the falling condition was calculated according to the Eq (14) [52–55]. It is worth noting that the members of the fall arrest system have to withstand such force.

$$F=mg\sqrt{1+(2E\cdot S\cdot f)/mg}$$ (14)

where,

F: impact force

m: mass

g: gravity acceleration 9.8 m/s^2^

E: elastic modulus

S: section of the rope

f: fall factor

The rope used for the tests was 10.5 *mm* in diameter, and the elastic modulus was 3.59• 10^8^ *N/m*^*2*^. The fall factor was expected to be almost 0, considering the fall distance was the same as the rope length. Based on these values, the impact force 1.61 *kN* was obtained.

**Fig 14:**
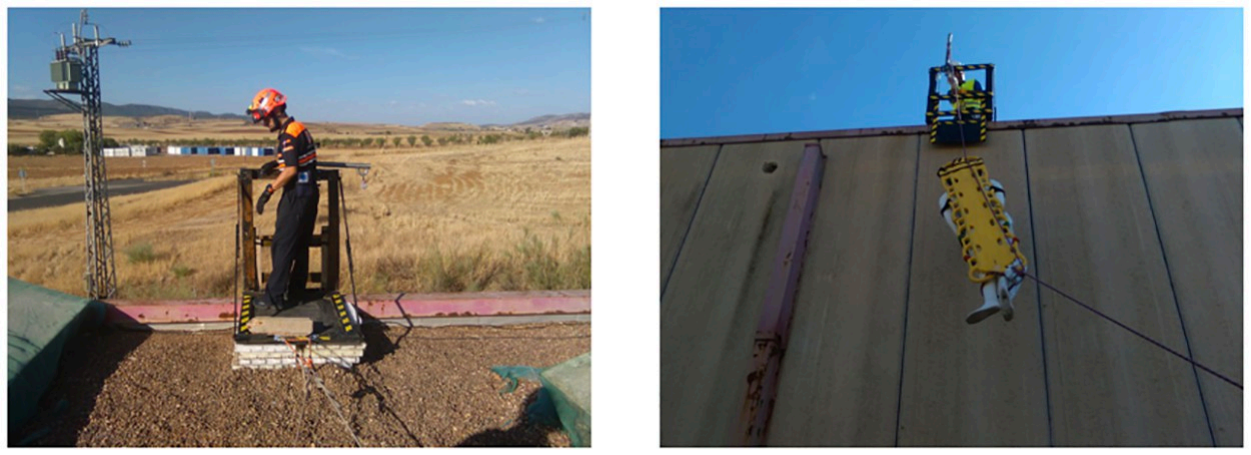
Accidental load test. (a) Before the test. (b) After the test.

Having determined the impact force, both the dummy and the TEPS were prepared for the test. The TEPS was adjusted to an inclination of 0, and securely fixed with two ropes to the structure of the building by using Class B anchors [56], which spread the force generated between both anchors, as shown in Fig 14a. After the test under the supervision of experts, no delamination, buckling, breakage and deformation were observed, see Fig 14b.

From the results of the field tests, it is obvious that the TEPS prototype can work well under static and accidental loading conditions. Moreover, it also substantially improves rescue in height operations and prevents the rescuer´s fall.

### Limitations of the new TEPS

The novel TEPS presented is not a substitute for the shoring system necessary to prevent a trench collapse, but rather a complement to them that prevents workers and objects from falling into the trench.

The system may be used without the trench being shored up only in the following cases as mentioned in the standard NTP 278 [6]:

- In the case of properly stabilized trenches with slopes.
- Excavations with cohesive soil and a depth of less than 1.30 meters without nearby solicitations.

Like the shoring system, the TEPS must be reviewed at the start of the work day and these preventions will be extreme after work interruptions of more than one day and/or atmospheric disturbances such as rain or frost.

## Conclusions

In the building and construction industry, trench work is a common activity, and the scenario for most of the fatal accidents or serious injuries owing to people or objects falling into trenches.

This novel edge protection system is effective as fall protection for different trench shoring systems and in buildings without edge protection systems, and consists of an edge barrier that functions as a conventional fall protection barrier that is attached to a platform by two lateral folding support hinges; and an inner sliding panel running on the guiderails of the edge barrier that enables trench shoring of the upper part of the trench wall. Moreover, assembly and installation of the system is fast, simple, and safe, with no need for workers to be on the edge of a trench.

The analyses of the Ultimate Limit State with fundamental load (ULS), the Ultimate Limit State with Accidental Load (AL), and the Serviceability Limit State (SLS) revealed the new edge protection system outperformed the conventional one.

Furthermore, the results of the validity and field tests of the prototype of the new temporal edge protection system show that it meets the requirement of the standard and stands the static and accidental loads. It can be used in preventing falls from heights.
